# Identification of Reproductive Trait-Associated Loci and Candidate Genes in Commercial Pigs via 50K SNP Genotyping and Genome-Wide Association Study

**DOI:** 10.3390/biology15100766

**Published:** 2026-05-11

**Authors:** Wenwu Chen, Fang Yang, Yantong Chen, Sui Liufu, Kaiming Wang, Zhi Li, Haiming Ma

**Affiliations:** 1 College of Animal Science and Technology, Hunan Agricultural University, Changsha 410128, China; 2 Key Laboratory of Livestock and Poultry Resources (Pig) Evaluation and Utilization, Ministry of Agriculture and Rural Affairs, Changsha 410128, China; 3 Yuelushan Laboratory, Changsha 410128, China; 4 Hunan Key Laboratory for Conservation and Utilization of Biological Resources in the Nanyue Mountainous Region, Hengyang Normal University, Hengyang 421008, China

**Keywords:** GWAS, ROH, reproduction, Yorkshire, candidate genes

## Abstract

To unravel the genetic basis of economically critical reproductive traits in swine, we genotyped 839 sows from three commercial breeds (Duroc, Landrace, Yorkshire) using the Porcine Breeding Chip_plus 50K SNP array, and analyzed three key traits: total number born (TNB), number born alive (NBA), and number of healthy piglets (NHP). We integrated principal component analysis (PCA) for population structure, runs of homozygosity (ROH) detection, genome-wide association studies (GWAS), and GO/KEGG enrichment analysis. Phenotypically, Yorkshire sows exhibited superior and persistent reproductive capacity across parities 1–7 (peak TNB: 14.17 ± 2.82 at parity 4 based on *N* ≥ 3 data), Duroc sows had limited data with only parity 1 available (TNB: 9.44 ± 2.13), and Landrace sows showed moderate to high performance across parities 1–4 and 7, with peak TNB at parity 4 (17.08 ± 4.61). ROH analysis further revealed that short ROH fragments (1–5 Mb) were the most abundant category across breeds, while the majority of detected ROH were under 10 Mb in length. GWAS identified significant SNPs concentrated on chromosomes 1 and 2, and annotated candidate genes including *AMH* (ovarian reserve), *IZUMO4* (embryo implantation), *ACSBG2* (steroid synthesis), *RFX2* (follicular maturation), and *DOT1L* (embryonic development). GO/KEGG enrichment highlighted pathways such as “histone methyltransferase activity” and “fatty acid biosynthesis”, which are critical for reproductive processes. This study clarifies breed-specific reproductive patterns and identifies key genetic loci/genes for porcine reproductive traits, providing molecular markers and a theoretical basis for improving swine reproductive performance via molecular breeding.

## Simple Summary

This study focused on unraveling the genetic underpinnings of three key economic reproductive traits in commercial pigs—total number born (TNB), number born alive (NBA), and number of healthy piglets (NHP). To this end, we genotyped 839 sows from three major commercial breeds (Duroc, Landrace, and Yorkshire) using the Porcine Breeding Chip_plus 50K SNP array, and analyzed the data through a suite of integrated genomic tools, including population structure analysis, runs of homozygosity (ROH) detection, genome-wide association studies (GWAS), and functional enrichment analysis. Phenotypically, Yorkshire sows stood out with the most superior and persistent reproductive performance, reaching a peak TNB of 14.172 ± 2.817 at parity 4. Duroc sows showed limited data with only parity 1 available (TNB: 9.44), while Landrace sows exhibited moderate to high performance across parities 1–4 and 7, with peak TNB at parity 4 (17.08). GWAS identified significant genetic markers concentrated on chromosomes 1 and 2, and annotated potential candidate genes with reported roles in reproductive processes, such as *AMH* and *IZUMO4*. ROH analysis further revealed that short ROH fragments (1–5 Mb) constituted the most abundant category, a pattern that reflects the genetic characteristics of commercial pig populations. This work not only clarifies the breed-specific reproductive patterns of the three commercial pig breeds but also identifies core genetic loci and genes associated with porcine reproductive traits. Importantly, it provides practical molecular markers to facilitate the improvement of pig reproductive performance through molecular breeding strategies.

## 1. Introduction

Reproductive traits, particularly litter size (total number born, TNB; number born alive, NBA; and number of healthy piglets, NHP), are among the most economically critical traits in the global swine industry. As a major driver of production efficiency, improving litter size directly enhances the output of commercial pig farms and contributes significantly to the sustainability of livestock supply chains [1]. However, porcine reproductive traits are complex quantitative traits controlled by multiple genes (quantitative trait loci, QTLs) and strongly influenced by environmental factors, making their genetic dissection a long-standing challenge in animal breeding [2]. In recent decades, the advent of high-throughput genotyping technologies has revolutionized the study of complex traits, with genome-wide association studies (GWAS) emerging as a powerful tool for identifying genetic variants and candidate genes underlying phenotypic variation. Compared to traditional linkage mapping, GWAS offers higher resolution by leveraging historical recombination events across populations, enabling the efficient localization of causal or tightly linked genetic markers to reproductive trait-related QTLs at the genome scale. Consequently, GWAS has been widely adopted to dissect the genetic basis of porcine reproductive traits over the past 15 years, with a growing body of literature reporting candidate genes and genomic regions associated with litter size and related traits across diverse pig breeds [3].

Among the most well-characterized candidates are genes with established roles in reproductive physiology, such as Estrogen Receptor 1 (*ESR1*), Follicle-Stimulating Hormone Beta Subunit (*FSHβ*), and Bone Morphogenetic Protein Receptor Type 1B (*BMPR1B*). *ESR1*—a key mediator of estrogen signaling—was first identified as a major QTL for litter size in Chinese Meishan pigs, where a missense mutation was associated with a 0.5–1.0 increase in TNB [4]; subsequent studies confirmed its association with reproductive performance in commercial breeds like Large White and Landrace, albeit with varying effect sizes [5]. *FSHβ*, which encodes the beta subunit of follicle-stimulating hormone (a central regulator of folliculogenesis and ovulation), has been linked to increased ovulation rate in Yorkshire pigs and improved NBA in local breeds such as the Rongchang pig [6]. *BMPR1B*, as a critical component of the *BMP* signaling pathway that governs follicle development and granulosa cell differentiation, was found to be associated with litter size in Duroc pigs and Chinese indigenous breeds like the Jinhua pig [7]. These genes have become foundational in understanding the genetic architecture of porcine reproduction and have even been explored for marker-assisted selection (MAS) in early breeding programs.

However, despite these advances, the practical application of these GWAS-derived findings in commercial breeding remains limited, primarily due to pronounced variability in results across studies. A key contributor to this inconsistency is the divergence in breed genetic background: commercial pig breeds have undergone intense artificial selection for growth and lean meat percentage, leading to reduced genetic diversity in reproductive trait-related regions compared to Chinese indigenous breeds, which are naturally selected for high fecundity [8]. This genetic differentiation results in breed-specific allele frequencies of QTLs; *ESR1* mutation associated with high litter size in Meishan pigs is present at a low frequency in Duroc pigs, explaining why its effect is not detectable in Duroc-focused GWAS [9]. Additionally, sample size limitations often compromise the statistical power of GWAS: small cohorts are prone to false-positive associations or failure to detect QTLs with small effect sizes, which are common for complex traits like litter size [10]. For instance, a study with 300 Yorkshire sows identified *FSHβ* as a significant candidate for TNB, while a larger study (*n* = 1200) using the same breed failed to replicate this association, likely due to improved statistical rigor in the latter [11].

Duroc, Landrace, and Yorkshire are the most widely raised commercial pig breeds globally, known for their distinct reproductive characteristics. For instance, Yorkshire sows are recognized for their high and persistent litter size, while Duroc sows often show breed-specific reproductive performance patterns across parities [12]. Exploring the genetic basis of reproductive trait differences among these breeds can provide valuable insights into breed-specific genetic mechanisms and facilitate the development of targeted breeding strategies.

Moreover, runs of homozygosity (ROH), which reflect genomic regions with consecutive homozygous genotypes, are closely related to inbreeding, genetic drift, and selection pressure [13]. Integrating ROH analysis with GWAS can help clarify the impact of population genetic structure and selection history on reproductive traits, enhancing the accuracy of candidate gene identification. Functional enrichment analysis of candidate genes further reveals the biological pathways underlying reproductive performance, bridging the gap between genetic variants and phenotypic variation [14].

In this study, we selected 839 sows from three commercial breeds (Duroc, Landrace, and Yorkshire) and systematically analyzed three key reproductive traits: TNB, NBA and NHP. Using the PorcineBreedingChip_plus 50K SNP array, genotyping, population structure analysis, and ROH detection were conducted to characterize the genetic background of the population. GWAS were performed to identify significant SNPs associated with the target traits, followed by annotation of candidate genes and functional enrichment analysis using GO and KEGG databases. The objectives of this study are as follows: (1) to explore the genetic variants and candidate genes underlying reproductive traits in the three commercial breeds; (2) to reveal the biological functions and pathways involved in regulating sow reproductive performance; (3) to provide theoretical basis and molecular markers for improving pig reproductive traits through molecular breeding.

## 2. Materials and Methods

### 2.1. Experimental Animals and Sampling

The experimental animals in this study were all sourced from a commercial intensive pig farm (Xinwufeng Co., Ltd., Changde, China) under consistent feeding and management regimens. A total of 839 farrowing sows across three breeds (a total of 19 Duroc pigs, 646 Yorkshire pigs, and 174 Landrace pigs were included in the study) were included. Three phenotypic traits (NBA, NHP, TNB) were statistically summarized and used for subsequent analyses. Blood samples were collected from all 839 sows, and genomic DNA was extracted via the magnetic bead method. DNA concentration and quality were assessed using a NanoDrop 2000 spectrophotometer (Thermo Scientific, Waltham, MA, USA) and agarose gel electrophoresis. For electrophoresis, 1 × TAE running buffer and TaKaRa DNA markers (Takara Biotechnology (Dalian) Co., Ltd., Dalian, Liaoning, China) were used, with a BIO-RAD DYPC-31BN system (Beijing Liuyi Biotechnology Co., Ltd., Beijing, China) for gel separation and a Newbio Gi-1 device for imaging (New Bio Co., Ltd., Gwangju, South Korea). All 839 samples met the quality requirements for genotyping with the Porcine Breeding Chip_plus 50K (Beijing Kangpusen Biotechnology Co., Ltd., Beijing, China).

### 2.2. Phenotypic Traits

Three phenotypic traits (NBA, NHP, and TNB) were statistically summarized and used for subsequent analyses. Descriptive statistical analysis of phenotypes was performed using R software (Version 4.2.1) to assess data quality. The statistical indicators included mean, standard deviation, coefficient of variation, as well as the minimum and maximum values of each trait. The normal distribution characteristics of phenotypes and the correlation between different phenotypic values were visually analyzed using the “GGally” package (Version 2.2.1), with simultaneous calculation of correlation coefficients.

### 2.3. Genotyping and Quality Control

Quality control of genotype data was conducted using PLINK software (Version 1.90 beta) to filter for high-quality shared SNPs across the three breeds (Duroc, Landrace, Yorkshire) [15]. The specific filtering criteria were as follows: (1) Individual call rate > 90%; (2) Minor Allele Frequency (MAF) > 5%; (3) *p*-value of Hardy–Weinberg Equilibrium (HWE) test > 10^−6^. In addition, SNPs located on sex chromosomes and mismatched regions were excluded. After quality control, 57,466 SNPs and 839 individuals were retained. The retained SNPs yield an approximate density of 1 SNP per 52 kb across the autosomal genome (~2.45 Gb, Sscrofa11.1). These QC thresholds follow established PLINK defaults widely validated in livestock GWAS. Finally, Beagle v5.2 software [16] was employed to impute missing genotypes. Given the low post-QC missing data rate and the haplotype-based algorithm of Beagle v5.2, imputation quality is expected to be high for 50K array data.

### 2.4. Runs of Homozygosity Detection

In this study, the R package detectRUNS (version 0.9.6) was utilized to identify ROH segments across autosomal chromosomes for each pig breed (Duroc, Landrace, and Yorkshire) using a sliding window approach. To accurately define an ROH and minimize false-positive segments caused by linkage disequilibrium or genotyping errors, specific criteria and thresholds were strictly applied: (1) a minimum ROH length of 1 Mb; (2) a sliding window of 50 SNPs; (3) a minimum of 50 consecutive SNPs included in an ROH; (4) a minimum density of at least one SNP per 100 kb within each ROH; (5) a maximum allowed gap of 1 Mb between consecutive SNPs; and (6) a maximum of five missing genotypes and one heterozygous genotype permitted per sliding window. Following ROH identification, the total number and genomic length of the ROHs were calculated for each individual. To comprehensively characterize the homozygosity landscape, the detected segments were subsequently classified into three distinct length-based categories: 1–5 Mb, 5–10 Mb, and >10 Mb.

To pinpoint genomic regions exhibiting a remarkably high frequency of homozygosity across the populations, ROH islands were determined independently for each breed by extracting the top 1% of SNPs most frequently observed within ROH segments. Overlapping high-frequency SNPs were then merged to establish the precise boundaries of these ROH islands. The genomic coordinates of the identified islands were aligned to the Sus scrofa reference genome (assembly Sscrofa11.1) to retrieve annotated genes using the Ensembl database via the biomaRt package in R (Version 2.60.0). Finally, to elucidate the biological functions and metabolic pathways associated with these highly homozygous regions, Gene Ontology (GO) and Kyoto Encyclopedia of Genes and Genomes (KEGG) enrichment analyses were conducted. Statistical significance for the functional enrichment was established using the Benjamini–Hochberg (BH) method for multiple testing correction, applying strict significance cutoffs of *p* < 0.05 and Q < 0.20.

### 2.5. Population Structure and Kinship Identification

To assess population structure among the three breeds (Duroc, Landrace, Yorkshire) and control for potential stratification in subsequent GWAS, PCA was performed on the high-quality genotype data of all 839 individuals. Eigenvalues and eigenvectors were calculated using GCTA software (Version 1.94.1) to quantify genetic variation among individuals. The first two principal components (PC1 and PC2) were visualized using the “ggplot2” package in R (Version 4.2.1) to illustrate genetic clustering of the three breeds. Additionally, a genomic relationship matrix (G-matrix) was constructed via GCTA for kinship identification, and a mixed linear model (MLM) was used to correct for population stratification in GWAS.

### 2.6. Genome-Wide Association Study (GWAS)

GWAS was conducted with rMVP (version 1.3.0) to examine associations of each SNP with phenotypic data. The linear mixed model used was y = Zα + Wb + g + e, where y stands for the vector of phenotypic values of each individual; α refers to the vector of fixed effects, which include variety, parity and the top five eigenvectors from principal component analysis; Z is the indicator matrix corresponding to α; W represents the marker matrix; and b is the respective marker effect to be tested; we also used g~N (0, Mσ_g^2^), where M is used to describe the genetic association between individuals, and σ_g^2^ represents the genetic variance of the polygenic effect; likewise, e~N (0, Iσ_e^2^) indicates the residual error, where I is the identity matrix and σ_e^2^ is the residual variance.

Multiple testing was corrected using the Bonferroni method. Based on the total number of filtered SNPs, the genome-wide significance threshold was set at 8.7 × 10^−7^ (calculated as 0.05/N), and the genome-wide suggestive threshold was set at 1.74 × 10^−5^ (calculated as 1/N). The λ values for three different phenotypes are: TNB: λ = 0.9143; NBA: λ = 0.9459; NHP: λ = 0.9242. The λ values (0.9143–0.9459) are consistent with effective confounding control in this multi-breed cohort, as confirmed by QQ plot calibration.

### 2.7. Enrichment Analysis of Candidate Genes

The candidate genes were identified based on significant SNPs using gene annotation databases (Sus scrofa genome (Sscrofa11.1)). Genes located within a certain physical distance of the significant SNPs were considered candidate genes (100 kb window centered on each genome-wide significant SNP), and redundant genes were removed to form the final candidate gene list. Background gene set was defined as all genes annotated in the reference genome (all protein-coding genes in Sscrofa11.1) to avoid bias caused by incomplete gene sets.

Functional enrichment analysis was performed to explore the biological significance of candidate genes, including the following. Gene Ontology (GO) enrichment, focusing on three categories: Biological Process (BP), Cellular Component (CC), and Molecular Function (MF). Kyoto Encyclopedia of Genes and Genomes (KEGG) pathway enrichment: analyzing the involvement of candidate genes in known metabolic or signaling pathways. The analysis was conducted using online tool DAVID (v6.8). The hypergeometric test was used to calculate the significance of enrichment for each term/pathway.

## 3. Results

### 3.1. Description of Phenotypes

After completing the phenotypic recording of the 839 sows, to obtain genomic data for subsequent genetic association analysis, all sows were genotyped using the Illumina Porcine SNP 50K BeadChip (Illumina, San Diego, CA, USA); raw genotyping data were further filtered (call rate > 90%, MAF > 0.05, HWE *p*-value > 1 × 10^−6^) to ensure quality. There were 57,466 SNPs loci in total. The reproductive performance of Landrace, Duroc, and Yorkshire sows across parities was analyzed. For Duroc, only parity 1 met the *N* ≥ 3 threshold (9.444 ± 2.128 TNB, 7.667 ± 2.121 NBA, 7.556 ± 2.128 NHP, *n* = 9), precluding cross-parity comparisons. Landrace showed moderate to high performance across parities 1–4 and 7, with peak TNB at parity 4 (17.077 ± 4.609), though sample sizes were limited for later parities (*n* = 13 at parity 4, *n* = 3 at parity 7). Yorkshire sows exhibited superior and sustained reproductive capacity across parities 1–7 (*N* ≥ 3), with TNB peaking at parity 4 (14.172 ± 2.817, *n* = 58), and NBA/NHP also remaining high even at higher parities. Overall, Yorkshire showed the most robust and persistent performance across multiple parities, while Duroc data were insufficient for cross-parity analysis and Landrace showed promising but limited late-parity data (Table 1). Given the biological interdependence of TNB, NBA, and NHP, we quantified their phenotypic correlations: TNB-NBA (r = 0.882), TNB-NHP (r = 0.842), and NBA-NHP (r = 0.964; all *p* < 0.001).

### 3.2. Characteristics of Runs of Homozygosity (ROH) Distribution in the Studied Population

Across all three breeds, short ROH fragments (1–5 Mb) constituted the most abundant category, representing 43.8%, 56.2%, and 49.9% of total ROH segments in Duroc, Landrace, and Yorkshire, respectively. Medium (5–10 Mb) and long (>10 Mb) ROH accounted for the remainder, with long ROH contributing disproportionately to total ROH length (35.4–55.0%) despite their lower segment count (13.6–22.5%; Supplementary Materials S2). Genome-wide ROH coverage ranged from 8.90% (Landrace) to 31.64% (Duroc). We characterized the landscape of ROH across three pig breeds (Supplementary Materials S3–S5). A consistent pattern emerged across all breeds, with chromosome 1 (SSC1) identified as the primary hotspot for homozygosity, exhibiting the highest average ROH count and the largest proportion of genomic coverage. This suggests that SSC1 harbors key loci under intense artificial selection for economically important traits.

**Figure 1:**
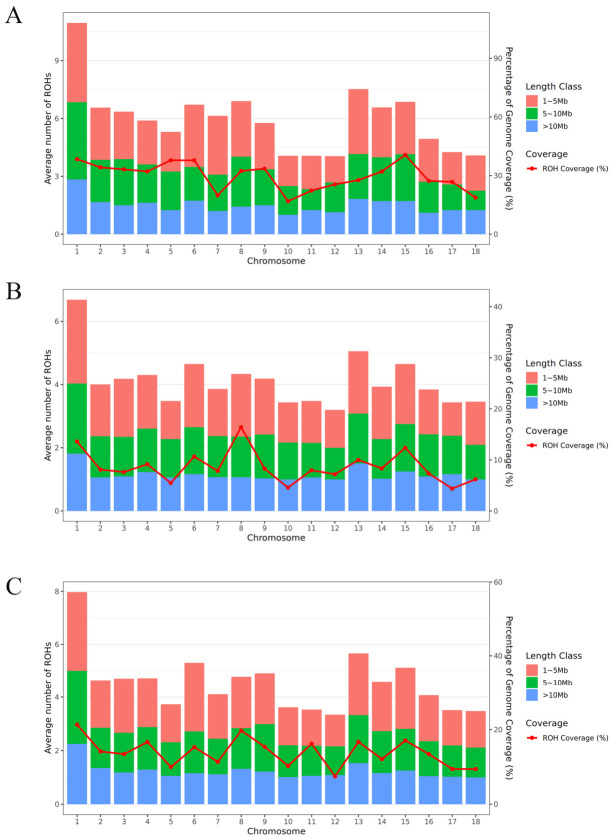
The frequency distribution of the average number of ROHs per chromosome (bars) and average percentage of each chromosome covered by ROHs (lines). (**A**) Duroc (**B**) Landrace, and (**C**) Yorkshire.

Notably, distinct breed-specific patterns were observed. The Duroc breed exhibited the highest genome-wide ROH coverage (31.64%) and presented an anomalously high density of ROH on SSC1 (>10 segments; Figure 1A). However, given the limited sample size, this extreme signal likely reflects the influence of specific highly inbred families or founder effects within the sampled cohort rather than a definitive breed-wide characteristic. Further validation with a larger Duroc population is warranted to distinguish between sampling bias and inherent breed features.

In contrast, Landrace pigs exhibited a more balanced genomic homozygosity profile. While SSC1 remained prominent, ROH segments were more uniformly distributed across multiple chromosomes, including SSC6, SSC8, SSC13, and SSC15 (Figure 1B). This dispersed pattern may reflect the breeding history of Landrace as a maternal line, where selection pressure is balanced across multiple traits rather than focused on a single production trait, thereby preserving greater genomic diversity outside of core selected regions.

Finally, the Yorkshire population displayed a pattern of extreme localized homozygosity. Despite having the largest sample size, which typically normalizes population variance, Yorkshire pigs showed the highest average ROH count on SSC1 (~8.0 segments; Figure 1C), with a total genome-wide ROH coverage of 14.84%. Their ROH distribution followed a “single-peak dominant” model, heavily concentrated on SSC1 and SSC13. Furthermore, the enrichment of long ROH segments (>10 Mb) on SSC1 indicates significant recent inbreeding and strong directional selection targeting specific genomic regions in this breed (Figure 1C).

**Figure 2:**
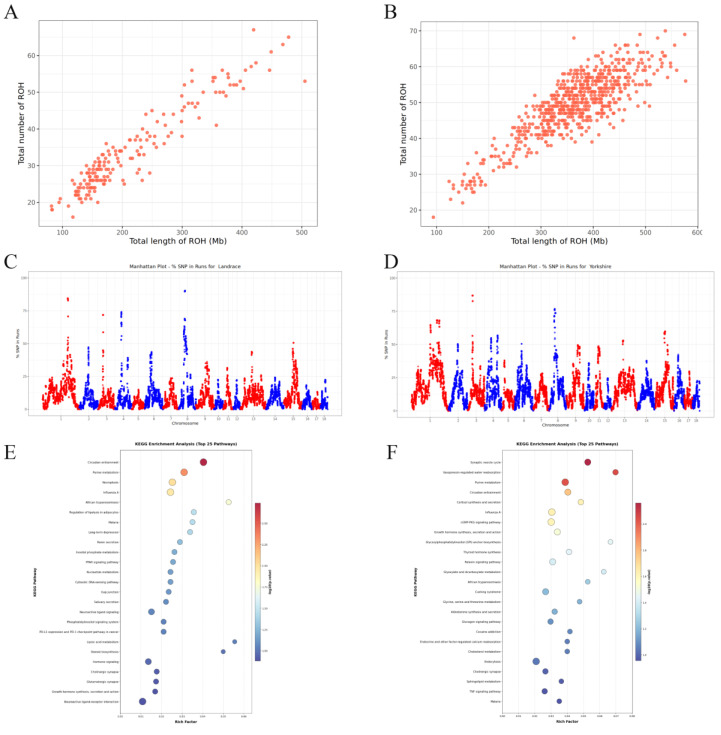
Genomic landscapes of ROH reveal divergent selection strategies in maternal pig breeds (**A**,**B**) Scatter plots showing the correlation between the number of ROH segments and total ROH length per individual for Landrace (**A**) and Yorkshire (**B**) pigs. (**C**,**D**) Manhattan plots displaying the percentage of SNPs within ROH regions across the genome for Landrace (**C**) and Yorkshire (**D**). (**E**,**F**) Top enriched KEGG pathways for genes located within ROH regions in Landrace (**E**) and Yorkshire (**F**).

Genomic analysis of ROH revealed distinct selection signatures underlying reproductive performance in the two maternal breeds, Landrace and Yorkshire; results for the Duroc breed were excluded from the main text due to limited sample size and are provided in Supplementary Materials S6. Among the primary maternal lines, while both breeds exhibited a strong positive correlation between ROH number and total length, Landrace displayed a more homogeneous inbreeding distribution compared to the highly variable pattern observed in Yorkshire (Figure 2A,B). Chromosomal mapping uncovered divergent “selection hotspots”: Landrace showed a dispersed multi-peak profile with significant ROH enrichment on SSC6, SSC8, and SSC15 (Figure 2C), whereas Yorkshire exhibited an extreme, concentrated fixation primarily on SSC1 and SSC13 (Figure 2D). This structural divergence was functionally corroborated by KEGG enrichment analysis; Landrace ROH were significantly associated with PPAR signaling pathways, regulation of lipolysis pathways (Figure 2E), indicative of genetic optimization for mobilizing energy to support large litters. In contrast, Yorkshire ROH were enriched for growth hormone synthesis pathways and cortisol secretion pathway (Figure 2F), reflecting a selection strategy focused on enhancing maternal robustness, hormonal regulation. Together, these results highlight two unique genomic architectures for maternal efficiency: a metabolism-driven strategy for high fecundity in Landrace versus an endocrine-driven strategy for robustness and growth in Yorkshire.

### 3.3. GWAS

**Figure 3:**
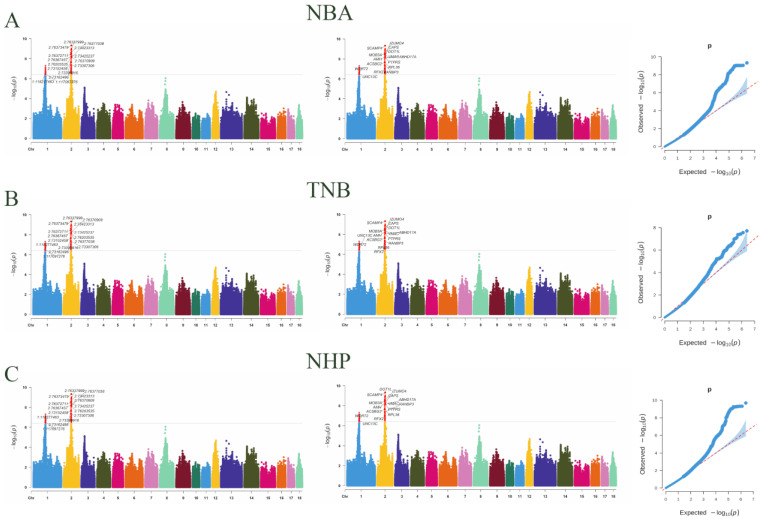
Manhattan plots and quantile-quantile (QQ) plots illustrating the NBA (**A**), the TNB (**B**), and the NHP (**C**). The horizontal dashed lines in the Manhattan indicate the suggestive level (8.7 × 10^−7^). The QQ plots show the observed −log10-transformed *p*-values (y-axis) and the expected −log10-transformed *p*-values (x-axis). The λ values for three different phenotypes are as follows: TNB: λ = 0.9143; NBA: λ = 0. 9459; NHP: λ = 0.9242.

Analysis of the three traits reveals that all the significant loci are concentrated on Chromosome 1 and Chromosome 2 (65 genome-wide significant SNPs in NBA; 55 genome-wide significant SNPs in TNB; 86 genome-wide significant SNPs in NHP, Supplementary S7). Meanwhile, annotation of all SNPS reveals that the related genes are mainly: *SCAMP4*, *IZUMO4*, *ACOT11*, *MOB3A*, *AMH*, *PTPRS*, *ACSBG2*, *ZNF83*, *WFIKKN1*, *RFX2*, *ANXA3*, *UNG13C*, etc. (Figure 3A). Genes such as *UNC13C*, *WDR72*, *MLLT1*, *ACSBG2*, *MOB3A*, *AMH*, *PTPRS*, *ACSBG2*, *ZNF83*, *WFIKKN1*, *RFX2*, and *VMAC* may be key candidate genes associated with the trait of NHP (Figure 3B). Genes such as *ACSBG2*, *RFX2*, *NRTN*, *HSD11B1L*, *LONP1*, *RPL36*, *PTPRS*, *DOT1L*, *AMH*, *IZUMO4*, *MOB3A*, and *SCAMP4* may be key candidate genes associated with the trait of TNB. Notably, three candidate genes—AMH, RFX2, and PTPRS—were consistently identified across all three traits (TNB, NBA, NHP), indicating their potential role as core regulators of overall reproductive performance (Figure 3C).

### 3.4. Enrichment Analysis

**Figure 4:**
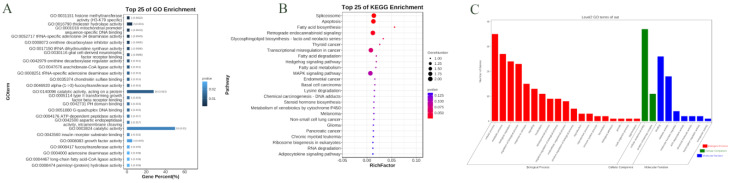
GO and KEGG enrichment analyses were conducted on the differentially screened genes. Top 25 of GO enrichment (**A**). Top 25 of KEGG enrichment (**B**). Level 2 GO enrichment. (**C**) GO Level 2 functional classification of DEGs, showing gene counts for Biological Process (red), Cellular Component (green), and Molecular Function (blue) categories.

To gain a deeper understanding of the association between the functions of these related genes and reproductive traits, GO and KEGG enrichment analyses were conducted on the genes. The GO results showed that the main enriched pathways included “GO:0031351 histone methyltransferase” and “GO:0001018 mitochondrial promoter sequence-specific DNA binding”. “GO:2000355 negative regulation of ovarian follicle development” (Figure 4A,C). KEGG pathway enrichment indicated that most genes were enriched in “Spliceosome”, “Fatty acid biosynthesis”, “Apoptosis” (Figure 3B, Supplementary Materials S8–S10).

## 4. Discussion

### 4.1. Breed-Specific Differences in Reproductive Performance

Phenotypic analysis revealed distinct reproductive patterns among the three breeds: Yorkshire sows exhibited superior and persistent reproductive capacity across parities 1–7 (based on *N* ≥ 3 data), with peak performance at parity 4; Duroc sows had insufficient sample sizes for cross-parity analysis (only parity 1 met *N* ≥ 3 threshold, *n* = 9), precluding conclusions about parity-specific patterns; and Landrace data, though limited in sample size for later parities, showed peak performance at parity 4 (17.08 TNB) and moderate performance at parity 1 (13.49 TNB). These findings align with previous reports highlighting breed-specific reproductive traits: Yorkshire is widely recognized for high litter size and reproductive longevity, attributed to its long-term selection for maternal performance 4, while Duroc, traditionally selected for growth and meat quality, often exhibits lower but more parity-dependent reproductive efficiency 5. The parity-specific performance of Duroc may reflect genetic trade-offs between growth and reproduction, a phenomenon observed in other meat-type breeds. Landrace data, though limited for later parities, showed peak performance at parity 4 (17.08 TNB), suggesting potential for high fecundity in later parities that warrants further investigation with expanded sample sizes.

### 4.2. Runs of Homozygosity (ROH) and Population Genetic Structure

ROH analysis revealed a right-skewed distribution, with most ROHs being short (1–5 Mb), likely reflecting genetic drift, while longer ROHs may result from inbreeding or selection. The positive correlation between total ROH length and quantity suggests cumulative effects of inbreeding or historical selection pressure on the genome. Short ROH enrichment is consistent with the outbred nature of commercial pig populations, where intensive selection limits long-term homozygosity [17].

Analysis of pathway enrichment in Landrace revealed that fatty acid desaturase 1 (*FASD1*) and Apolipoprotein F (*APOF*) were concurrently enriched in both the PPAR signaling pathway and the lipolysis regulation pathway. In contrast, pathway enrichment analysis in Yorkshire pigs identified Estrogen receptor 1 (*ESR1*) and Forkhead box A1 (*FOXA1*) as significantly associated with the growth hormone synthesis pathway and the cortisol secretion pathway.

*FADS1* is a key rate-limiting enzyme in the synthesis of polyunsaturated fatty acids, and mainly regulates lipid metabolism through the PPAR signaling pathway [18,19]. The PPAR pathway is critical for follicular development and steroid hormone synthesis in sows. *FADS1* affects the ligand activity of PPAR by altering fatty acid composition, thereby maintaining ovarian lipid metabolic homeostasis [20]. Abnormal lipolysis leads to free fatty acid accumulation in follicles, causing granulosa cell apoptosis and reduced oocyte quality, ultimately impairing reproductive performance. In this study, *FADS1* was located in ROH-enriched regions, indicating that this gene has undergone targeted selection for reproductive traits in Yorkshire sows and can serve as an important candidate gene for sow fertility.

*APOF* is mainly involved in lipoprotein metabolism and lipolysis regulation, maintaining physiological lipid homeostasis [21]. During the reproductive cycle of sows, ovarian cholesterol supply and lipolytic balance directly determine progesterone synthesis, follicle maturation, and corpus luteum function [22]. Dysfunction of *APOF* causes disturbed lipolysis and imbalanced follicular fluid lipids, reducing ovulation rate and embryo survival [23]. The significant ROH signal in the *APOF* region in this study suggests that this gene participates in the regulation of reproductive traits in Yorkshire sows via the lipolysis regulation pathway, acting as an important candidate gene linking lipid metabolism and reproduction.

*ESR1* is a key regulator of sow reproductive performance, and its association with litter size has been established as a major quantitative trait locus in pigs. Rothschild et al. reported that a specific *ESR1* allele was associated with increased litter size: in synthetic sows (50% Meishan background), homozygotes for the favorable allele produced 2.3 more piglets in first parity and 1.5 more across all parities; in Yorkshire ancestry, the favorable allele added more than one pig per litter. Subsequent studies confirmed the value of marker-assisted selection using ESR1 in Large-White-based lines [24], with other work estimating additive substitution effects of approximately 0.40 piglets per litter [25]. Although the precise mechanisms are still under investigation, *ESR1* is known to play critical roles in the hypothalamic–pituitary–gonadal axis, ovarian and endometrial function, placental angiogenesis, and mammary gland development [26]. Together, these findings support the integration of *ESR1* genotyping into marker-assisted selection programs for swine breeding.

Forkhead box A1 (*FOXA1*), a pioneer transcription factor involved in chromatin remodeling and nuclear hormone receptor signaling, was identified as a candidate gene for sow reproductive traits in the present study. *FOXA1* acts as a pioneer factor that opens compacted chromatin for other proteins through interactions with nucleosomal core histones, and it modulates the transcriptional activity of nuclear hormone receptors, including *ESR1*. *FOXA1* is involved in ESR1-mediated transcription and is required for *ESR1* binding to target gene promoters [27]. Genome-wide association studies have identified numerous QTLs and candidate genes associated with sow reproductive traits such as TNB and NBA, highlighting the complex polygenic architecture underlying litter size [28,29]. Additionally, studies have systematically identified candidate genes related to sow litter size and teat number through GWAS combined with functional pathway analysis, revealing a complex transcriptional factor network regulating reproductive traits [30]. Functional studies have shown that *FOXA1* participates in the differentiation of porcine ovarian granulosa cells and the formation of corpora lutea, indicating its potential role in ovarian function [31]. In summary, *FOXA1*, through its pioneer factor activity and synergy with *ESR1*, may affect sow reproductive performance by regulating ovarian function and reproductive-related transcriptional networks.

Notably, population structure PCA showed stratification among breeds and within Yorkshire, emphasizing the need for PCA correction in GWAS to avoid false associations—a critical step validated by our QQ plots showing well-controlled population stratification. Integrating ROH with GWAS helps distinguish trait-associated loci from those shaped by neutral processes, enhancing candidate gene reliability [32].

### 4.3. Genetic Loci and Candidate Genes Associated with Reproductive Traits

GWAS identified significant SNPs concentrated on Chromosomes 1 and 2, with annotation revealing candidate genes such as *IZUMO4*, *AMH*, *PTPRS*, *ACSBG2*, and *RFX2*. These genes have been previously implicated in reproductive processes in mammals, and their proximity to significant SNPs suggests potential involvement in porcine reproductive traits. Anti-Mullerian Hormone (*AMH*), a member of the *TGF-β* superfamily has been established as critical for ovarian follicle development in mammals, and its association with TNB/NHP here suggests a potentially conserved role in pigs and is widely used as a marker of ovarian reserve in mammals [33]. Studies have shown that when the *AMH* content is high, the probability of oocyte fertilization is higher, and at the same time, the ratio of high-quality embryos is higher and the probability of embryo development and maturity is also higher [34]. The research by Shiori Saito KOHIGASHI et al. found that in the reproductive process of cattle, cows with higher *AMH* levels had a larger ovarian reserve capacity and a greater number of small follicles, indicating that *AMH* can be used as an indicator for evaluating the ovarian reserve of cattle [35]. Our identification of *AMH* associated with TNB and NHP supports its conserved role in regulating follicle maturation and ovulation rate, consistent with GWAS findings in other pig breeds [36].

*IZUMO4* encodes a soluble glycoprotein featuring a conserved *IZUMO* domain at its N-terminus, which is capable of forming dimers [37]. Members of the *IZUMO* family mediate membrane fusion through specific binding to oocyte surface receptors such as *JUNO* [38]. In pigs, *IZUMO1* exhibits marked heterogeneity in localization—for instance, a punctate distribution in the equatorial region—whereas *IZUMO4* may enhance binding stability via heterodimer formation. The establishment of endometrial receptivity hinges on the synergistic action of multiple secreted proteins, and the detection of *IZUMO4* in uterine secretions suggests it may facilitate embryo implantation based on its known role in membrane fusion and detection in uterine secretions by regulating the expression of adhesion molecules like integrins [39]. Its association with NBA here underscores potential roles in successful fertilization and early embryonic survival, representing a novel insight that broadens our understanding of reproductive molecular mechanisms beyond folliculogenesis.

The receptor tyrosine phosphatase encoded by the *PTPRS* gene comprises an extracellular immunoglobulin-like domain, a transmembrane region, and an intracellular catalytic domain [40]. Its core function lies in regulating cellular signaling pathways via reversible phosphorylation. In human endometrial cells, *PTPRS* modulates trophoblast cell adhesion by regulating the phosphorylation status of integrin β3. *PTPRS* is highly expressed in placental trophoblast cells and may sustain maternal-fetal immune tolerance through inhibiting T cell activation [41]. *PTPRS* was enriched in the apoptosis pathway. Apoptosis regulation potentially influences embryonic development and placental function; aberrant apoptosis can lead to fetal loss [42], suggesting *PTPRS* may influence NHP by modulating cell survival during early gestation.

Acyl-CoA Synthase Bubblegum Family Member 2 (*ACSBG2*), a core member of long-chain fatty acid-activating enzymes, encodes a protein featuring a conserved AMP-binding domain. This domain enables the protein to catalyze the conversion of long-chain and very long-chain fatty acids into active acyl-CoA derivatives, which in turn provide substrates for lipid synthesis and β-oxidation [43]. Notably, the synthesis of ovarian steroid hormones relies on the supply of lipid precursors [44]. The fatty acids activated by *ACSBG2* can act as raw materials for cholesterol synthesis in granulosa cells, thereby influencing the follicle-stimulating hormone (*FSH*)-induced proliferation and differentiation of these cells [45].

Regulatory Factor X2 (*RFX2*) belongs to the *RFX* transcription factor family. The protein it encodes contains a conserved winged-helix DNA-binding domain and may contribute to gene expression by binding to the X-box element in the promoter region of target genes [46]. This gene is highly evolutionarily conserved. The porcine *RFX2* gene is located on chromosome 2, consists of 19 exons, and encodes a 728-amino-acid protein. It shares over 85% homology with its counterparts in mammals such as humans and mice [47]. Studies have demonstrated that in mouse ovarian granulosa cells, *RFX2* has been reported to regulate estrogen synthesis and follicular maturation in mouse models, suggesting a potentially analogous function in porcine reproduction by regulating the expression of *CYP19A1* (aromatase) and *STAR* (steroidogenic acute regulatory protein) genes [48]. It is therefore speculated that *RFX2* may regulate follicular selection and ovulation number in pigs through the same mechanism.

Through GO enrichment analysis, it was found that the *DOT1L* gene was enriched in the “Histone methyltransferase activity” and “negative regulation of ovarian follicle development” pathway, which is crucial for oocyte maturation and embryonic development [49]. Previous studies have confirmed that after treating porcine nuclear transfer (*SCNT*) embryos with the *Dot1L* inhibitor EPZ004777, not only did the blastocyst rate significantly increase, but the level of histone H3 lysine 79 dimethylation (*H3K79me2*) also dropped to a level comparable to that of in vitro fertilization embryos. This suggests that *Dot1L*-mediated *H3K79* methylation might be one of the key obstacles in the *SCNT* embryo reprogramming process [50]. It can be inferred from this that the *DOT1L* gene can affect the reproductive traits of sows through the “Histone methyltransferase activity” and “negative regulation of ovarian follicle development” pathway.

Recent studies in cattle have expanded the analytical framework for understanding reproductive genomic architecture beyond conventional GWAS signals. Nayak et al. [51] proposed that genomic regions controlling reproductive fitness may exhibit periodic mathematical patterns amenable to detection through whole-genome sequencing, suggesting that deeper genomic order may underpin apparent GWAS signals. Complementarily, Nayak et al. demonstrated that selection signature analysis across indicine, taurine, and crossbred cattle can reveal breed-specific adaptive strategies for reproductive traits, with genes such as *GDF9* and *PRDM16* showing convergent selection pressures [52]. While our study utilized a 50K SNP array rather than whole-genome sequencing, our integration of ROH detection with GWAS similarly aims to capture the interplay between selection history and reproductive loci. Future work employing higher-density genotyping or sequence-based approaches could explore whether Fibonacci-like genomic regularities or additional selection signatures exist in commercial pig populations, as suggested by these cattle studies.

### 4.4. Limitations and Future Directions

This study has several limitations: First, Landrace data were restricted to parity 1, limiting inter-parity comparisons; expanding data to later parities would clarify breed-specific parity patterns. Second, candidate gene functions were inferred from annotations and enrichment, requiring functional validation to confirm their roles in reproductive traits. Third, while 50K SNPs provided genome coverage, higher-density arrays or whole-genome sequencing could refine locus resolution.

Future work should focus on the following: (1) Multi-omics integration to unravel gene regulatory networks in target tissues; (2) Cross-breed validation of candidate markers to assess their utility in diverse genetic backgrounds; (3) Longitudinal studies linking ROH dynamics to reproductive decline with parity, informing inbreeding management strategies.

## 5. Conclusions

From the perspective of genetic analysis, there are significant differences in the reproductive patterns of the three breeds, which are closely related to their genetic backgrounds: the high reproductive persistence of the Yorkshire pig under multiple parity confirms the genetic stability of maternal traits in its long-term breeding; The limited Duroc data (only parity 1 available) precluded analysis of parity-specific patterns, highlighting the need for expanded sampling in this terminal sire breed; ROH analysis further revealed that short ROH fragments (1–5 Mb) were the most abundant category across breeds, while the majority of detected ROH were under 10 Mb in length reflecting the breeding practice effectiveness of commercial pig breeds in controlling inbreeding and maintaining basic genetic diversity under high-intensity breeding. The integration analysis of GWAS and functional enrichment further clarified the core regulatory mechanism of reproductive traits: significant SNPs on chromosomes 1 and 2, as well as candidate genes such as *AMH*, *IZUMO4*, *ACSBG2*, connect key physiological pathways such as ovarian reserve, embryo implantation, and steroid synthesis, confirming that these genes represent promising candidate nodes for cross-breed regulation of pig reproductive performance, pending experimental validation of their causal effects. In summary, this study not only clarified the breed specific genetic basis of reproductive traits in commercial pig breeds, but also screened key markers and genes that can be directly applied to molecular breeding, providing precise directions for customized breeding strategies for different breeds.

## Figures and Tables

**Table 1:** Summary statistics of reproduction traits in the sow population.

Pig Breeds	Parity	*N*	TNB	NBA	NHP
Landrace	1	71	13.493 ± 2.699	11.676 ± 2.897	11.296 ± 2.748
	2	45	13.644 ± 3.142	12.022 ± 3.159	11.644 ± 3.016
	3	33	14.091 ± 2.566	12.000 ± 2.681	11.333 ± 2.558
	4	13	17.077 ± 4.609	15.000 ± 4.262	13.846 ± 4.018
	7	3	14.000 ± 1.732	12.000 ± 1.732	11.000 ± 1.000
Duroc	1	9	9.444 ± 2.128	7.667 ± 2.121	7.556 ± 2.128
Yorkshire	1	250	13.336 ± 2.519	11.500 ± 2.683	10.996 ± 2.486
	2	169	13.680 ± 2.292	11.763 ± 2.325	11.473 ± 2.166
	3	109	13.817 ± 2.746	12.156 ± 2.465	11.633 ± 2.222
	4	58	14.172 ± 2.817	12.483 ± 2.624	11.707 ± 2.384
	5	22	12.636 ± 3.064	11.045 ± 2.853	10.545 ± 2.464
	6	10	13.900 ± 2.234	12.200 ± 1.814	11.600 ± 1.350
	7	5	13.600 ± 3.912	11.200 ± 2.588	10.400 ± 3.286

Note: Values are shown as mean ± standard deviation. Only parities with *N* ≥ 3 are included for biological relevance. TNB = total number born, NBA = number born alive, NHP = number of healthy piglets. *N* = number of genotyped sows contributing to that parity. Phenotypic distribution of the three reproductive traits (TNB, NBA, NHP) across all sows showed a near-normal pattern (Supplementary Figure S1A), with no extreme outliers after data cleaning. This confirmed the reliability of the phenotypic data for subsequent GWAS and ROH analyses. Population structure PCA was conducted on the three breeds used for sequencing. It can be seen that there is population structure stratification in the three breeds, and at the same time, Yorkshire pigs also have intra-breed structure stratification (Supplementary Figure S1B). Therefore, before conducting GWAS, PCA correction was performed on all sample results.

## Data Availability

All data generated or analyzed during this study are included in this published article.

## Supplementary Materials

The following supporting information can be downloaded at: https://www.mdpi.com/article/10.3390/biology15100766/s1, Supplementary Materials S1: The distribution of SNPs on autosomal chromosomes and the principal component analysis; Supplementary Materials S2: Descriptive statistics of three classes of runs of homozygosity; Supplementary Materials S3: Genes located within ROH regions associated with the number of piglets born alive; Supplementary Materials S4: Genes located within ROH regions associated with the number of healthy piglets; Supplementary Materials S5: Genes located within ROH regions associated with the total number of piglets born; Supplementary Materials S6: Scatter plots showing the correlation between ROH segments and total ROH length, Manhattan plots of SNPs within ROH regions, and top enriched KEGG pathways for Duroc; Supplementary Materials S7: ROH islands with annotated genes for three breeds; Supplementary Materials S8: Top enriched KEGG pathways for genes associated with healthy piglets; Supplementary Materials S9: Top enriched KEGG pathways for genes associated with piglets born alive; Supplementary Materials S10: Top enriched KEGG pathways for genes associated with total piglets born.
