# Supplementary material for: Identification of Reproductive Trait-Associated Loci and Candidate Genes in Commercial Pigs via 50K SNP Genotyping and Genome-Wide Association Study
Source: Biology (Basel). 2026 May 11;15(10):766. doi: 10.3390/biology15100766 (PMC13203925; doi:10.3390/biology15100766)
Supplement: Supplementary file 1 [file biology-15-00766-s001.zip › Supplementary materials 1.pdf]

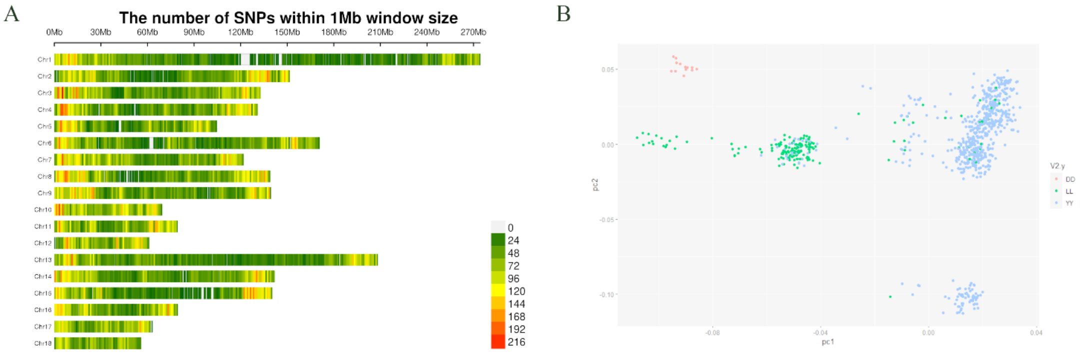

Supplementary Materials S1. The distribution of SNPs on autosomal chromosomes and the principal component analysis. (A) The number of SNPs within a 1 Mb window. (B) The PCA-plot of 3 breed pig principal components.
