# Supplementary material for: Identification of Reproductive Trait-Associated Loci and Candidate Genes in Commercial Pigs via 50K SNP Genotyping and Genome-Wide Association Study
Source: Biology (Basel). 2026 May 11;15(10):766. doi: 10.3390/biology15100766 (PMC13203925; doi:10.3390/biology15100766)
Supplement: Supplementary file 1 [file biology-15-00766-s001.zip › Supplementary materials 6.pdf]

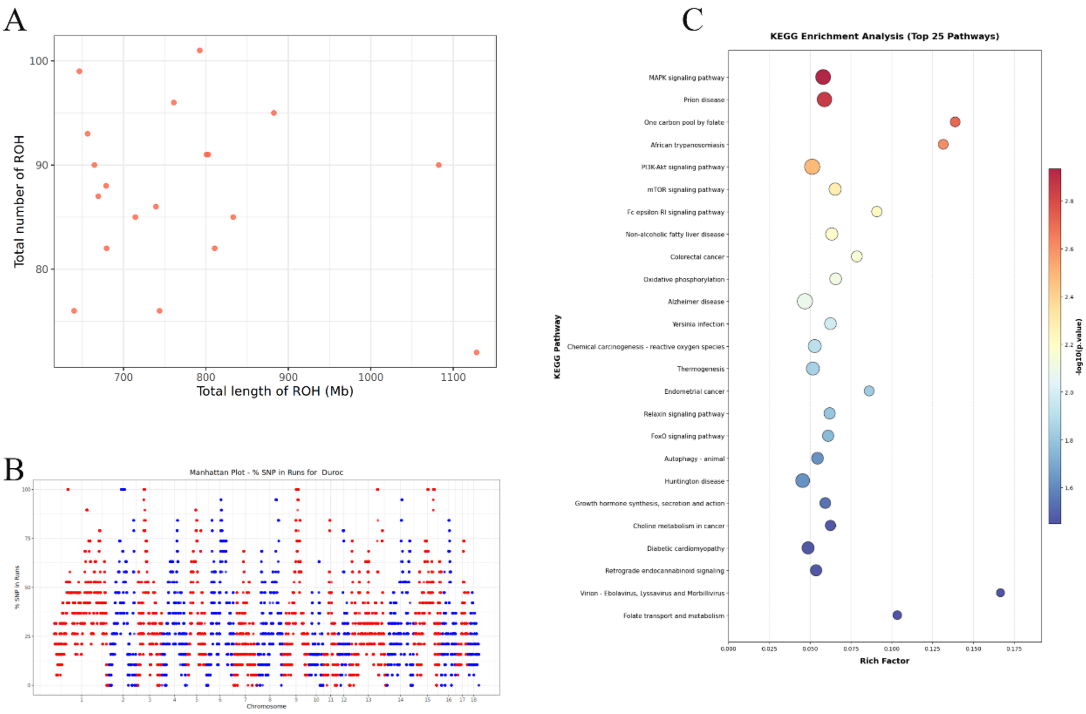

Supplementary materials S6: Scatter plots showing the correlation between the number of ROH segments and total ROH length per individual for Duroc (A) pigs. (B) Manhattan plots displaying the percentage of SNPs within ROH regions across the genome for Duroc. Top enriched KEGG pathways for genes located within ROH regions in Duroc (C).
